# Breed-related and stage-related differences in the immune response to *Staphylococcus aureus* carriage in sheep during the periparturient period

**DOI:** 10.2478/jvetres-2026-0043

**Published:** 2026-08-07

**Authors:** Agata Hahaj-Siembida, Aneta Nowakiewicz, Mariola Bochniarz, Aleksandra Trościańczyk, Marcelina Osińska, Andrzej Junkuszew, Karina Savvulidi Vargova, Monika Gregula-Kania

**Affiliations:** 1 Sub-Department of Veterinary Microbiology, Department of Preclinical Veterinary Sciences, University of Life Sciences in Lublin, 20-950 Lublin, Poland; 2 Department of Animal Breeding and Agricultural Consulting, University of Life Sciences in Lublin, 20-950 Lublin, Poland; 3 Institute of Pathological Physiology, First Faculty of Medicine, Charles University, 128 53 Prague, Czech Republic

**Keywords:** acute-phase response, cytokine gene expression, ewes, haptoglobin, IL6

## Abstract

**Introduction:**

*Staphylococcus aureus* is ubiquitous as a commensal bacterium and a frequent opportunistic pathogenic one in animals including livestock. The aim of this study was to analyse the expression levels of ovine cytokine genes and acute-phase proteins in response to the presence of *S. aureus*.

**Material and Methods:**

Four different anatomical swabs, milk and blood samples came from 50 ewes from the native Uhruska and Świniarka breeds. The Uhruska breed exhibits high fertility and meat performance and the Świniarka breed is considered a primitive breed. Isolation and identification of *S. aureus*, haematological analysis, and analysis of cytokine and acute phase protein gene expression levels were performed.

**Results:**

*Staphylococcus aureus* strains were isolated from swabs and milk from sheep of both breeds. A significant increase in *IL6* and *Hp* (haptoglobin) gene expression was observed in leucocytes in samples from Uhruska sheep in which the presence of *S. aureus* was detected.

**Conclusion:**

The expression level of selected cytokine and acute-phase protein genes have shown that even local carriage of *S. aureus* in small ruminants can lead to a systemic immune response, manifested by increased expression of *IL6* and *Hp* genes in leucocytes.

## Introduction

Coagulase-positive *Staphylococcus aureus* occurs naturally in animals and humans, but can also be responsible for numerous infections including mastitis in cattle, goats and sheep (15). These infections can negatively impact sheep and their lambs during the perinatal period, when they cause stress and pain in sheep and may ultimately cause the death of lambs and ewes (10). *Staphylococcus aureus* can induce an immune response in a variety of ways which depend on the host’s health status. One of the more problematic periods when animals are more susceptible to infection is pregnancy. Numerous anatomical, physiological, endocrinological, metabolic and immunological changes take place to initiate and sustain gestation. Maternal immunomodulation in pregnancy is the consequence of a shift from a T-helper 1 (Th1) to a T-helper 2 (Th2) lymphocyte response. The Th1 response has been shown to promote phagocytosis and killing of intracellular pathogens, while the Th2 response stimulates B cell proliferation and antibody production (27). During pregnancy, other changes occur in the body’s physical barriers, *i.e*. the skin and mucous membranes, which can facilitate colonisation by potential pathogens or the proliferation of opportunistic bacteria (23). These adaptations of the body to pregnancy increase the likelihood of the development of infections. Understanding these changes is crucial in the design of effective strategies to prevent and treat infections during this critical period. Comprehensive understanding of perinatal physiological and pathological conditions also supports the condition and welfare of lambs, because these directly depend on the health of the mother during pregnancy, parturition and lactation. Infections caused by *S. aureus* in farm animals during these specific periods can lead to serious health and economic problems. This bacterium is particularly harmful to dairy cattle and small ruminants, mainly causing mastitis or skin and joint inflammation (18, 26). In sheep and goats, *S. aureus* is also responsible for serious reproductive problems, such as abortions and stillbirths. Infections can lead to pregnancy loss, which has a direct impact on the number of lambs in the herd and the herd’s general health. In turn, staphylococcal endometritis leads to reduced fertility, an increased number of insemination services needed for fertilisation and premature culling of animals.

*Staphylococcus aureus* has many factors that interfere with the host’s immune response, and its presence activates various types of defence mechanisms. These bacteria have the ability to invade professional phagocytes and persist intracellularly in them. The host cells include neutrophils, monocyte-derived macrophages and mammary epithelial cells. The cocci can also form small colony variants and promote biofilm formation, which diminishes the effect of antibiotic therapy in both humans and animals. One of the groups of proteins involved in the innate immune system are cytokines (14, 31). Natural killer cells recognise the pathogen and trigger an inflammatory response by expressing certain cytokines, *e.g*. TNF-α or IL-1β and IL-8; they recruit neutrophils and macrophages from the bloodstream to the site of inflammation. In addition, cytokines can also increase the phagocytic activity of macrophages and neutrophils (14, 28).

The main proinflammatory cytokines are interleukin IL-6, IL-1 and TNF-α. Interleukin-6 is also considered one of the most important cytokines capable of inducing the synthesis of various acute-phase proteins (APPs). The concentration of circulating APPs is species specific and related to the severity of the disorder and the degree of tissue damage in the organism. The level of certain APPs in serum can increase during inflammation, and these are termed positive APPs, and that of others can decrease, those correspondingly being termed negative APPs. These attributes convey diagnostic and prognostic information, thus making APPs useful biomarkers in monitoring animal health (21, 24).

*Staphylococcus aureus* can trigger the induction of gene expression by infected host cells that reflect activation of immune pathways. Assessing the sheep host’s immune response to intracellular *S. aureus* will help identify which elements of the ovine immune system are involved and which species-specific features can be noted. The aim of this study was to compare the expression of the cytokine genes *IL-1α, IL-1β, IL-6*, and *TNF-α* and acute-phase protein genes *SAA* (serum amyloid A), *Hp* (haptoglobin) and *Cp* (ceruloplasmin) in leucocytes in four groups of sheep. The groups were: Uhruska-breed ewes from which *S. aureus* was isolated, Uhruska ewes free of this bacterial species, and counterparts of the Świniarka breed. The samples tested were collected from animals in four different physiological statuses. Most previous studies on the immune response in ruminants have been based on the analysis of somatic cells in milk (1) or udder endothelial cells. In our study, the analyses were conducted uniquely based on blood leucocytes. Research on immune gene expression in sheep is very sparse compared to the available research in other species (cattle and pigs), and the potential mechanisms of correlation between the activation of inflammatory processes and the presence of *S. aureus* are still not fully understood (25, 29).

The aims of this study were threefold. It sought first to determine whether the presence of *S. aureus* in sheep significantly affects the expression of proinflammatory cytokine genes and acute-phase protein genes involved in the blood-leucocyte immune response. The research also assessed whether the immune response differs significantly between the Świniarka and Uhruska breeds of sheep. The third aim was to investigate whether the pregnancy or lactation state modulates the expression of immune response genes in interaction with *S. aureus* infection in sheep.

## Material and Methods

### Description of animals and collection of materials

This study was part of a larger experiment investigating the occurrence and epidemiological significance of *S aureus* in two local sheep breeds. The research material was collected from ewes from a farm belonging to the University of Life Sciences in Lublin (south-eastern Poland). The research was conducted under a certificate (licence No. 95/2023) from the Local Ethical Committee for Animal Experiments in Lublin. The samples came from two native breeds of sheep: Uhruska and Świniarka. The Uhruska sheep exhibits high fertility and favourable meat production traits, and the Świniarka is considered a primitive breed. The ewes were all 3 years old and second parity. Before the experiment, the ewes were examined by a veterinarian, and only healthy individuals without clinical signs of disease were selected.

### Collection of microbiological and blood samples

The research material consisted of swabs from four different sites (the skin, ears, anus and mouth), milk and blood. The swabs were collected four times: before pregnancy, early in pregnancy, at the end of pregnancy and after lambing in lactation. Milk was collected only at the last time of swabbing. The blood samples were collected three times: before pregnancy, at the end of pregnancy and after lambing in lactation. A total of 800 swabs, 200 blood samples and 50 milk samples comprised the study material. Swabs from all four sites and blood were collected from 25 ewes of each breed each time. Milk was also collected from 25 individuals of each breeds, and each milk sample was 5 mL. The swabs and milk were collected with a previously described procedure (9).

Blood samples were collected by jugular venepuncture into sterile vacuum tubes by a veterinarian during a routine examination. Samples were transported to the laboratory under refrigeration and arrived in a maximum of 3 h. Whole blood was centrifuged at 3,000 rpm for 3 min to separate the buffy coat, then 250 μL of the buffy coat was pipetted into a new tube with 750 μL of RiboEx LS (GeneAll General Biotechnology, Seoul, South Korea) and stored at -80°C until analysis. Blood samples taken at the beginning of pregnancy were run through complete blood counts on an automated Abacus Junior Vet (Diatron, Budapest, Hungary) haematological analyser using the impedance method. Each sample was counted in duplicate, and the mean of both counts was used for the statistical analysis.

*Staphylococcus aureus* was then isolated from swabs and milk samples according to the culture procedure described by Hahaj-Siembida *et al*. (10). The presence of *S. aureus* in all samples was confirmed and its identification made according to previously described procedure (9, 11).

Total RNA was isolated from five samples from each group (Uhruska carriers of *S. aureus* and non-carriers and Świniarka carriers and non-carriers) to make a total of 20 samples per sampling period. Total RNA isolation from blood leucocytes was performed using the Hybrid-R Blood RNA kit (GeneAll General Biotechnology) according to the manufacturer’s protocol. The obtained total RNA was resuspended in 30 μL of RNase-free water, and samples were analysed for their quality using a NanoDrop 2000 device (Thermo Fisher Scientific, Wilmington, DE, USA). The absorbance ratio of the samples was measured at wavelengths of 260/280 nm and 260/230 nm, and samples were considered to be of good quality when the A260: A280 nm was ~2.0.

The iScript cDNA Synthesis Kit (BioRad, Hercules, CA, USA) was used to synthesise complementary DNA (cDNA) using the isolated RNA as the template according to the manufacturer’s protocol. The reaction was initially carried out in a PTC-220 DNA Engine thermocycler (MJ Research, Waltham, MA, USA). The synthesised cDNA was stored at –20°C. The level of transcripts of the studied genes was determined with the real-time PCR method using a CFX96 instrument (BioRad). The reaction was performed according to the manufacturer’s protocol for the iTaq Universal SYBR Green Supermix (BioRad). To prepare the reaction mixture, 100 ng of cDNA, 300 nM of each primer pair and 10 μL of iTaq Universal Sybr Green Supermix were used and water was added to make the volume up to 20 μL. The material was pipetted into wells of a transparent 96-well plate, in which a negative control (reaction mixture without cDNA) was also placed to ensure the purity of the reagents and to exclude contamination. A series of dilutions of the template cDNA (1, 1 : 2, 1 : 4, 1 : 16 and 1 : 64) was also prepared to determine the efficiency of the reaction and the standard curve. Most sequences of primers targeting the genes were selected based on literature data: those for *IL1A, IL1B* and *TNF* were as used by Bonnefont *et al*. (2) and the sequence for *IL6* was adopted from Hadfield *et al*. (8); the sequences for *SAA, Hp* and *Cp* gene primers were designed using the Primer-BLAST (basic local alignment search tool) algorithm. The sequences of the primers are shown in Table 1. Amplification reactions were performed at the temperature selected for the given pair of genes in 40 cycles according to the manufacturer’s protocol. The temperature of primer annealing was ascertained in preliminary studies conducted on pooled cDNA from the blood of individuals from both groups using a temperature-gradient PCR reaction. The reference genes used – *ACTB* (actin beta), *GAPDH* (glyceraldehyde-3-phosphate dehydrogenase) and *RPL19* (ribosomal protein L19) – were selected based on previous studies conducted by Greguła-Kania *et al*. (7) and Mauffré *et al*. (17). The relative mRNA expression level of the genes was estimated based on the Pfaffel formula (22).

**Table 1.** Primer sequences of the analysed genes

Gene	Primer sequences (5' to 3')
Cytokine *IL1A*	F: GATGACCTGGAAGCCATTGC R: ATGTGCTGATCTGGGCTTGAT
Cytokine *IL1B*	F: TGAAGAGCTGCACCCAACAC R: GAGAAATCTGCAGCTGGATGTTT
Cytokine *TNF*	F: GAGCACCAAAAGCATGATCC R: GGCGATGATCCCAAAGTAGA
Cytokine *IL6*	F: TAACCACTCCAGCCACACAC R: GATAACCTTTGCGTTCTTTACCC
Acute-phase protein *SAA* (serum amyloid A)	F: AAGAGGACACATCCCCACAG R: TCCCTGGTCATACCCTTGAG
Acute-phase protein *Hp* (haptoglobin)	F: CTCCCTGAATGTGAGGCAGT R: TGAGGTTATGGTGGGAGACC
Acute-phase protein *Cp* (ceruloplasmin)	F: GGAAGGATGTTTGGGAACCT R: TGGTATGTCCCAGGGAAGAG
Reference *ACTB* (actin beta)	F: AGCGTGGCTACAGCTTCACC R: GGCCATCTCCTGCTCGAAGT
Reference *GAPDH* (glyceraldehyde-3-phosphate dehydrogenase)	F: GCTGACGCTCCCATGTTTGT R: TCATAAGTCCCTCCACGATGC
Reference *RPL19* (ribosomal protein L19)	F: CCCCAATGAGACCAATGAAATC R: CAGCCCATCTTTGATCAGCTT

### Statistical analysis

Repeated-measures ANOVA was used to assess the effects of the sampling time, groups (carriers/non-carriers of *S. aureus*) and their interaction on the gene expression in leucocytes. Levene’s test was used to determine the homogeneity of variance. When the effect of the factors was significant (P-value < 0.05), a post-hoc Tukey test was performed to determine the specific differences between the means. Student’s *t*-test was used for comparing haematological parameters in blood between groups (carriers/non-carriers of *S. aureus*).

## Results

A total of 61 *S. aureus* strains were isolated from Uhruska ewes and 37 were from Świniarka ewes. No *5. aureus* strains were detected in the samples taken from pregravid Świniarka sheep.

The means of all haematological parameters in the blood of ewes in both groups were in physiological value ranges (16, 30) (Table 2). There were no statistically significant differences between the blood of carriers of *5. aureus* and that of non-carriers.

The expression of the four cytokine genes and three acute-phase protein genes was detected in the leucocytes of sheep carrying *S. aureus* and those not carrying it (Table 3). In Uhruska ewes, the expression of the *IL6* gene was generally higher in the leucocytes of sheep in which *S. aureus* was detected (Table 3, Fig. 3). A statistically significant increase in *IL6* and *Hp* gene expression, fourfold for the *Hp* gene, was observed during pregnancy compared to expression before pregnancy in the leucocytes in carrier Uhruska sheep, and both genes’ expression also declined after lambing to a significant extent (Table 3, Fig. 6). A statistically significant difference in the *IL6* gene expression was also observed between the carriers and non-carriers of *S. aureus* in lactating Uhruskas.

**Table 2.** Haematological parameters as means ± SEM in the blood plasma of *S. aureus* carrier and non-carrier ewes of parity two at study entry

Parameter	Breed	*S. aureus* non-carriers	*S. aureus* carriers	P-value
WBC (10^3^/μL)	Uhruska	6.6 ± 1.8	5.8 ± 2.3	0.4
WBC (10^3^/μL)	Świniarka	7.7 ± 1.6	8.7 ± 2.2	0.3
LYM (10^3^/μL)	Uhruska	3.7 ± 0.9	3.2 ± 1.2	0.3
LYM (10^3^/μL)	Świniarka	4.2 ± 0.7	3.8 ± 1.6	0.6
GRA (10^3^/μL)	Uhruska	2.8 ± 1.0	2.6 ± 1.3	0.4
GRA (10^3^/μL)	Świniarka	3.4 ± 1.2	4.7 ± 1.9	0.1
	Uhruska	9.9 ± 0.6	9.7 ± 1.5	0.7
RBC (10^6^/μL)	Świniarka	8.2 ± 0.5	8.0 ± 1.2	0.8
HGB (g/dL)	Uhruska	12.3 ± 0.8	12.3 ± 2.0	0.9
HGB (g/dL)	Świniarka	10.5 ± 1.0	10.6 ± 1.3	0.8
HCT (%)	Uhruska	29.4 ± 0.7	29.9 ± 1.6	0.7
HCT (%)	Świniarka	27.9 ± 2.1	27.3 ± 1.7	0.2
MCV (fl)	Uhruska	29.7 ± 1.3	29.8 ± 1.1	0.6
MCV (fl)	Świniarka	32.2 ± 0.9	31.3 ± 3.7	0.5
MCH (pg)	Uhruska	10.4 ± 0.5	10.6 ± 0.4	0.4
MCH (pg)	Świniarka	11.0 ± 0.4	11.5 ± 0.7	0.2

**Table 3.** *In vivo* expression of selected cytokine and acute-phase protein genes in the leucocytes of *S. aureus* carrier and non-carrier ewes of parity two at study entry, expressed as means ±SD

Genes	Breed	Carriage of *S. aureus*	Before pregnancy	SD	During pregnancy	SD	After lambing in lactation	SD
Cytokine *IL1A*	Uhruska	Non-carrier	3.07	±2.3	1.09	±0.23	2.17	±1.5
Cytokine *IL1A*	Uhruska	Carrier	1.36	±0.55	2.36	±1.05	1.94	±1.2
Cytokine *IL1A*	Świniarka	Non-carrier	2.96	±2.3	1.98	±1.4	1.94	±0.7
Cytokine *IL1A*	Świniarka	Carrier	na	na	2.07	±1.2	1.48	±0.8
Cytokine *IL1B*	Uhruska	Non-carrier	1.14	±0.33	1.06	±0.4	2.13	±1.3
Cytokine *IL1B*	Uhruska	Carrier	1.38	±0.55	1.16	±0.62	3.20	±2.2
Cytokine *IL1B*	Świniarka	Non-carrier	1.02	±0.2	1.17	±0.35	1.33	±1.05
Cytokine *IL1B*	Świniarka	Carrier	na	na	1.17	±0.35	7.51	±5.3
Cytokine *IL6*	Uhruska	Non-carrier	1.15	±0.33	1.34	±0.72	0.38*	±0.26
Cytokine *IL6*	Uhruska	Carrier	1.70^a^	±0.72	4.46^b^	±2.3	1.51^a^*	±0.7
Cytokine *IL6*	Świniarka	Non-carrier	1.33	±0.71	1.37	±0.5	1.51	±0.65
Cytokine *IL6*	Świniarka	Carrier	na	na	2.18	±1.2	0.41	±0.24
Cytokine *TNF*	Uhruska	Non-carrier	1.54	±0.6	1.22	±0.42	3.58	±2.1
Cytokine *TNF*	Uhruska	Carrier	1.11	±0.3	1.61	±0.50	1.16	±0.4
Cytokine *TNF*	Świniarka	Non-carrier	1.11	±0.25	1.29	±0.45	2.80	±1.35
Cytokine *TNF*	Świniarka	Carrier	na	na	1.77	±0.8	0.45	±0.29
Acute phase protein *SAA* (serum amyloid A)	Uhruska	Non-carrier	1.62	±1.2	1.39	±0.9	0.90	±0.7
Acute phase protein *SAA* (serum amyloid A)	Uhruska	Carrier	1.20	±0.35	1.25	±0.9	1.24	±0.4
Acute phase protein *SAA* (serum amyloid A)	Świniarka	Non-carrier	2.21	±1.7	1.87	±1.2	2.01	±0.8
Acute phase protein *SAA* (serum amyloid A)	Świniarka	Carrier	na	na	1.09	±0.7	0.55	±0.3
Acute-phase protein Hp (haptoglobin)	Uhruska	Non-carrier	1.05	±0.35	1.17*	±0.35	0.39	±0.20
Acute phase protein *Hp* (haptoglobin)	Uhruska	Carrier	1.08^a^	±0.25	4.7^b^*	±2.2	1.22^a^	±0.45
Acute phase protein *Hp* (haptoglobin)	Świniarka	Non-carrier	1.57	±0.55	1.79	±0.9	1.79	±0.9
Acute phase protein *Hp* (haptoglobin)	Świniarka	Carrier	na	na	2.30	±0.9	0.54	±0.32
Acute-phase protein *Cp* (ceruloplasmin)	Uhruska	Non-carrier	1.57	±0.9	1.48	±0.6	4.51	±4.2
Acute phase protein *Cp* (ceruloplasmin)	Uhruska	Carrier	1.31	±0.4	6.11	±3.5	1.50	±0.42
Acute phase protein *Cp* (ceruloplasmin)	Świniarka	Non-carrier	1.55	±0.6	1.39	±0.45	3.65	±2.1
Acute phase protein *Cp* (ceruloplasmin)	Świniarka	Carrier	na	na	3.61	±2.4	3.52	±3.3

^a, b^ – values in a row with different subscript letters are significantly different (P-value < 0.05);

* – values are significantly different between non-carriers and carriers (P-value < 0.05)

The figures below (Figs 1–7) present a graphical representation of cytokine and acute-phase protein genes expression in Świniarka and Uhruska sheep at the examined periods.

**Fig. 1.**
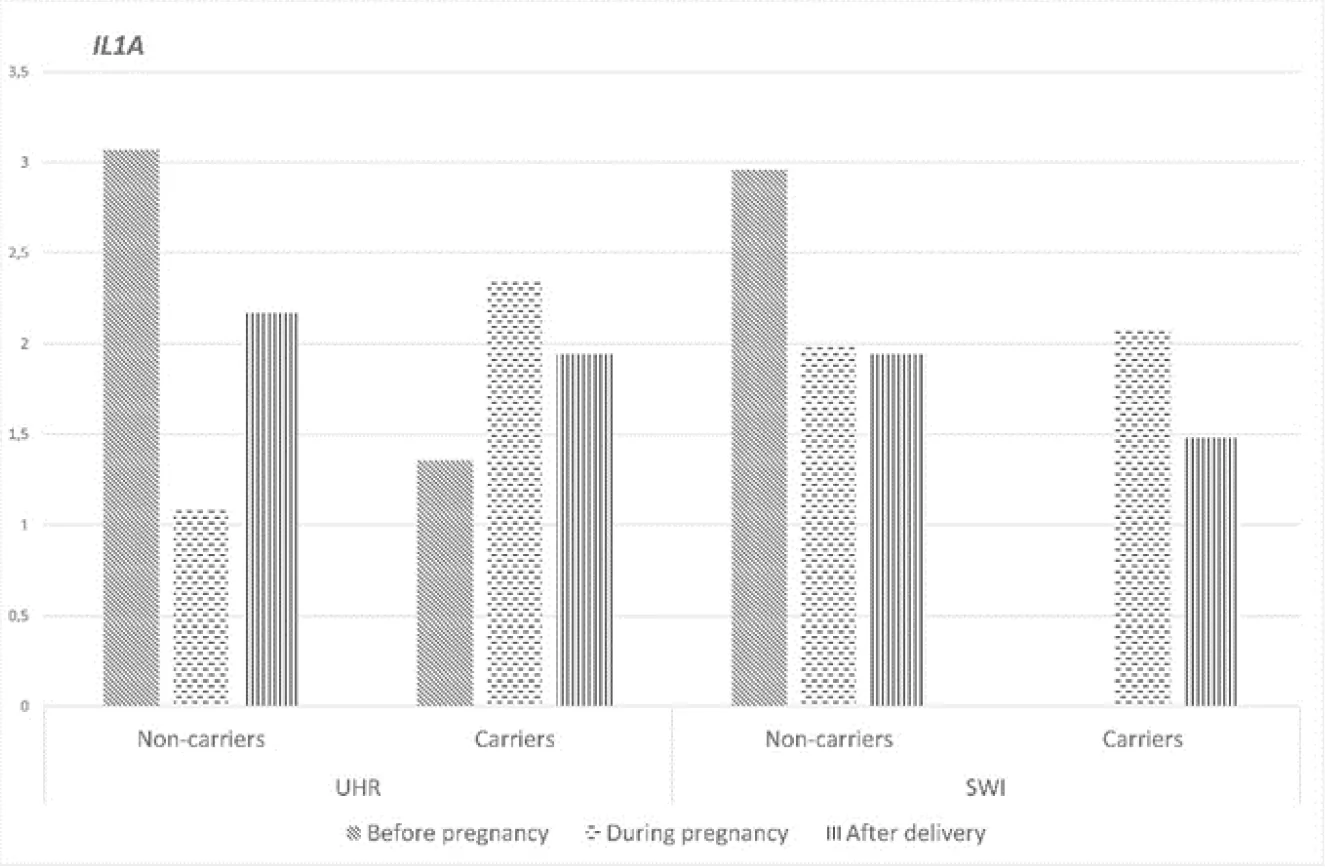
*IL1A* gene expression at different reproductive stages in Uhruska (UHR) and Świniarka (SWI) ewes of parity two at study entry

**Fig. 2.**
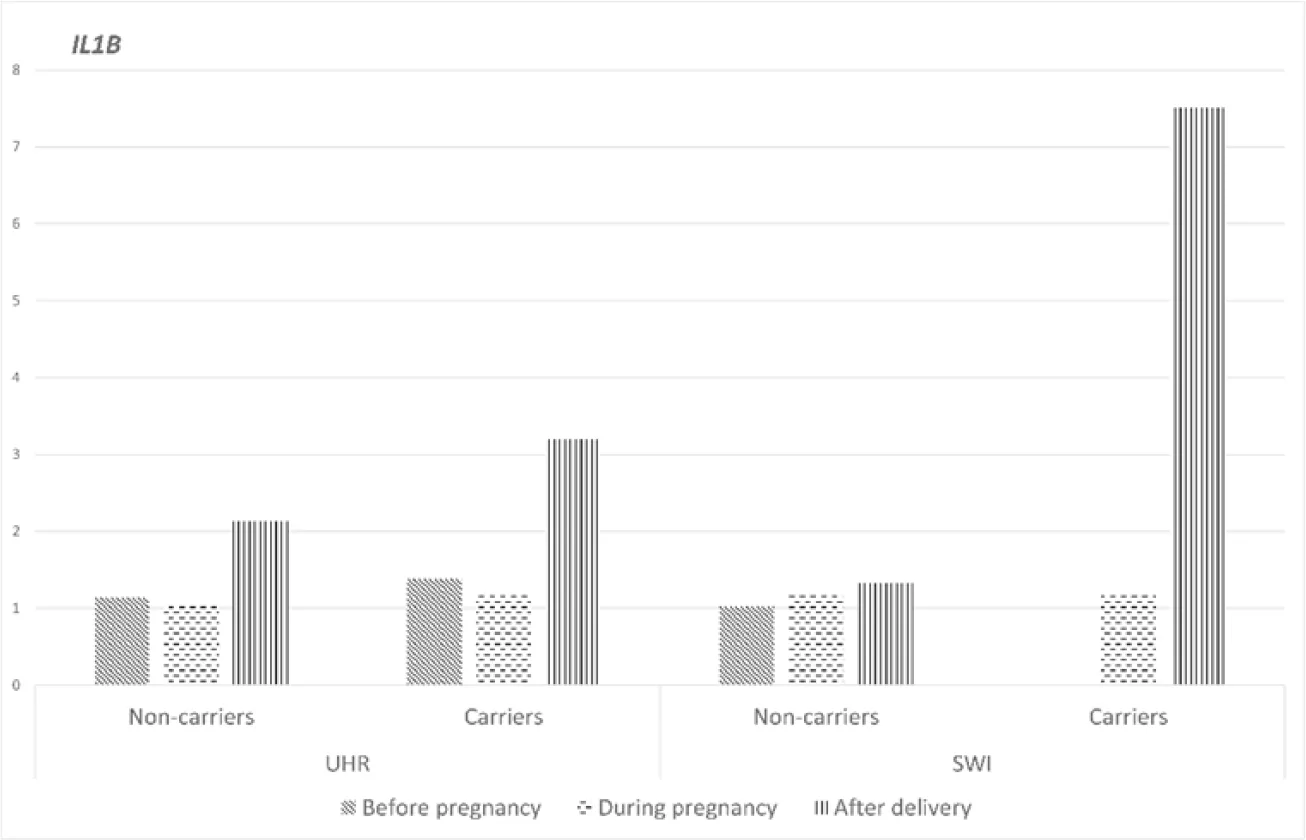
*IL1B* gene expression at different reproductive stages in Uhruska (UHR)and Świniarka (SWI) ewes of parity two at study entry

**Fig. 3.**
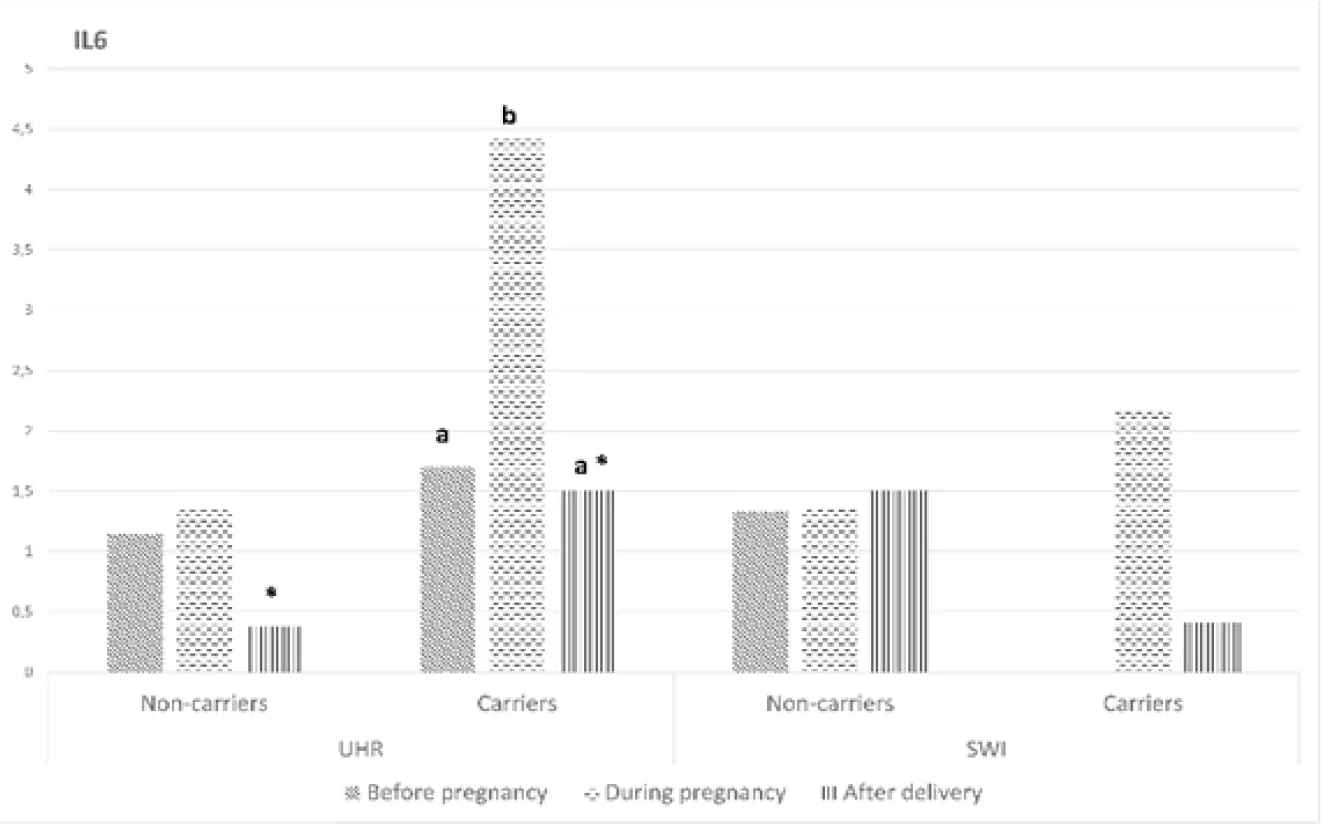
*IL6* gene expression at different reproductive stages in Uhruska (UHR) and Świniarka (SWI) ewes of parity two at study entry. Values represent the mean. ^a, b^ – means within each three-reproductive-stage group of bars with different subscript letters are significantly different (P-value < 0.05); * – means of non-carriers are significantly different to means of carriers (P-value < 0.05)

**Fig. 4.**
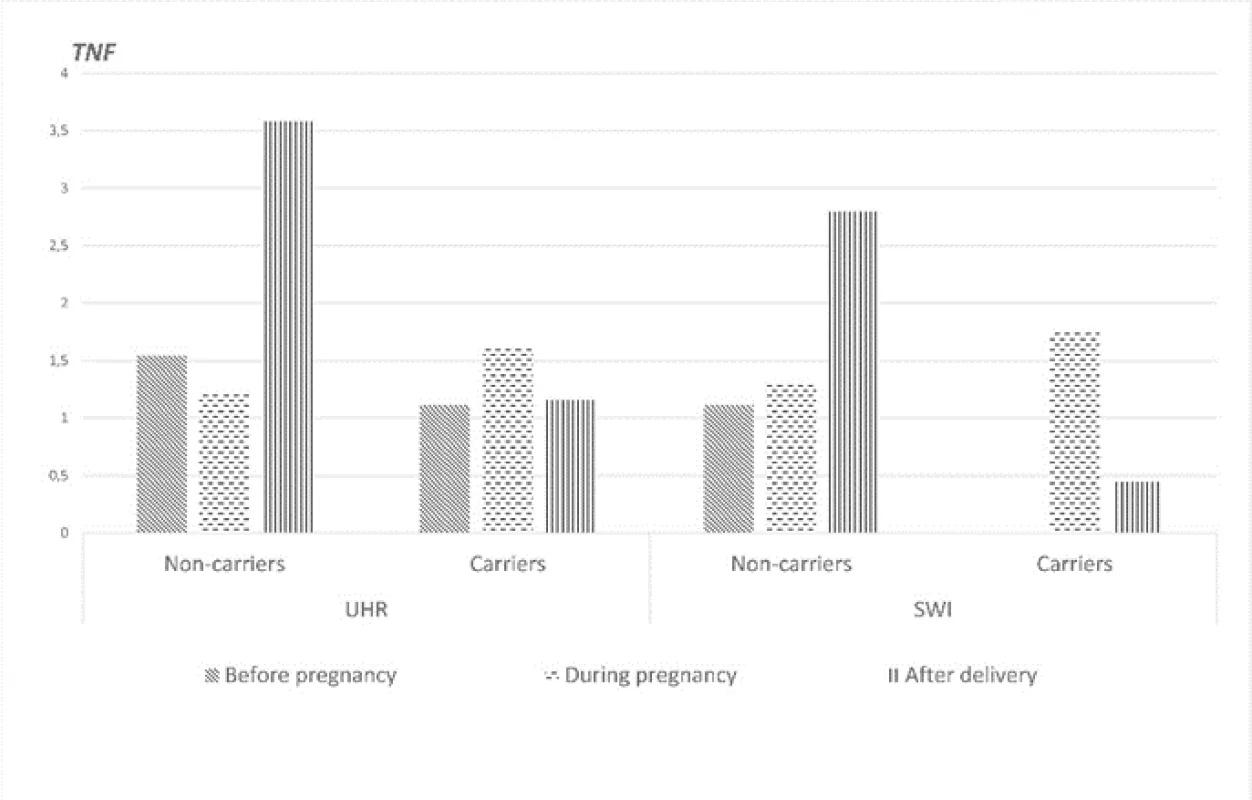
*TNF* gene expression at different reproductive stages in Uhruska (UHR) and Świniarka (SWI) ewes of parity two at study entry. Values represent the mean

**Fig. 5.**
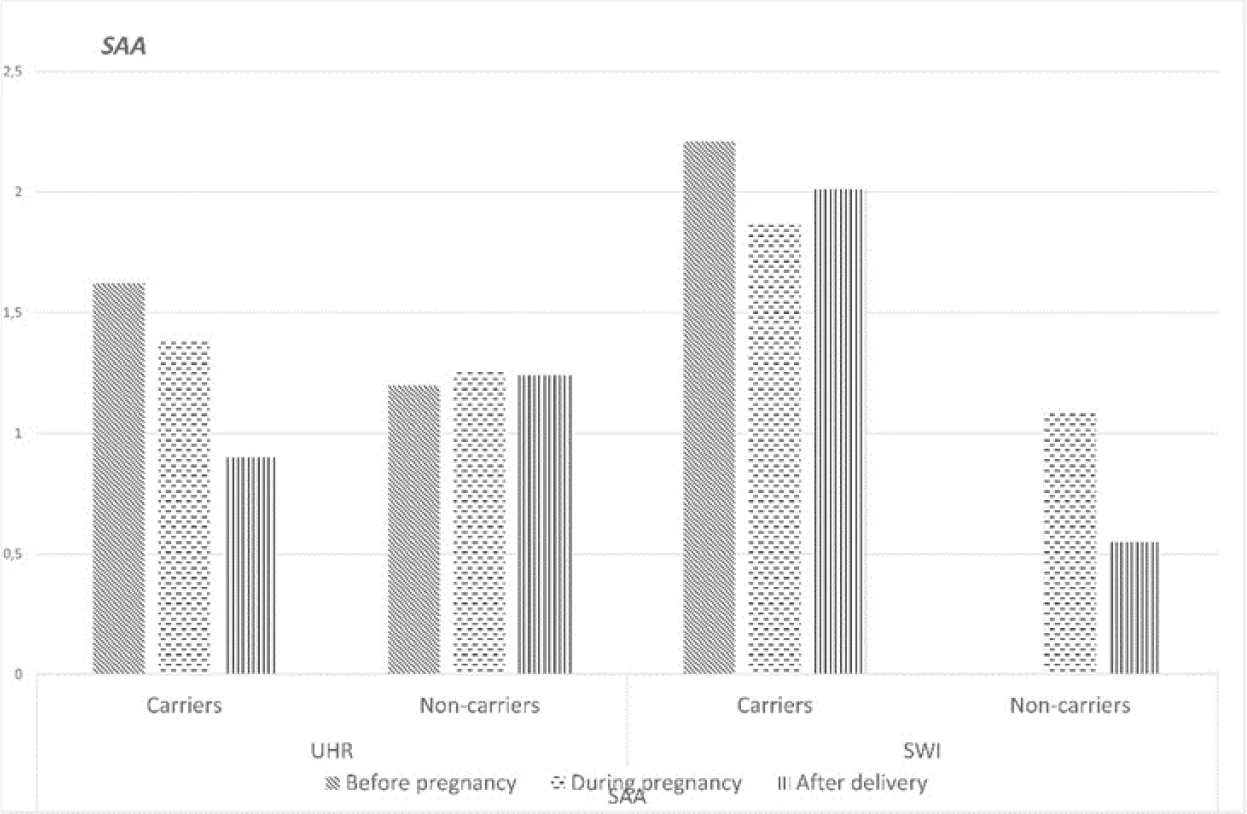
*SAA* gene expression at different reproductive stages in Uhruska (UHR) and Świniarka (SWI) ewes of parity two at study entry. Values represent the mean. ^a, b^ – means within each three-reproductive-stage group of bars with different subscript letters are significantly different (P-value < 0.05); * – means of non-carriers are significantly different to means of carriers (P-value < 0.05)

**Fig. 6.**
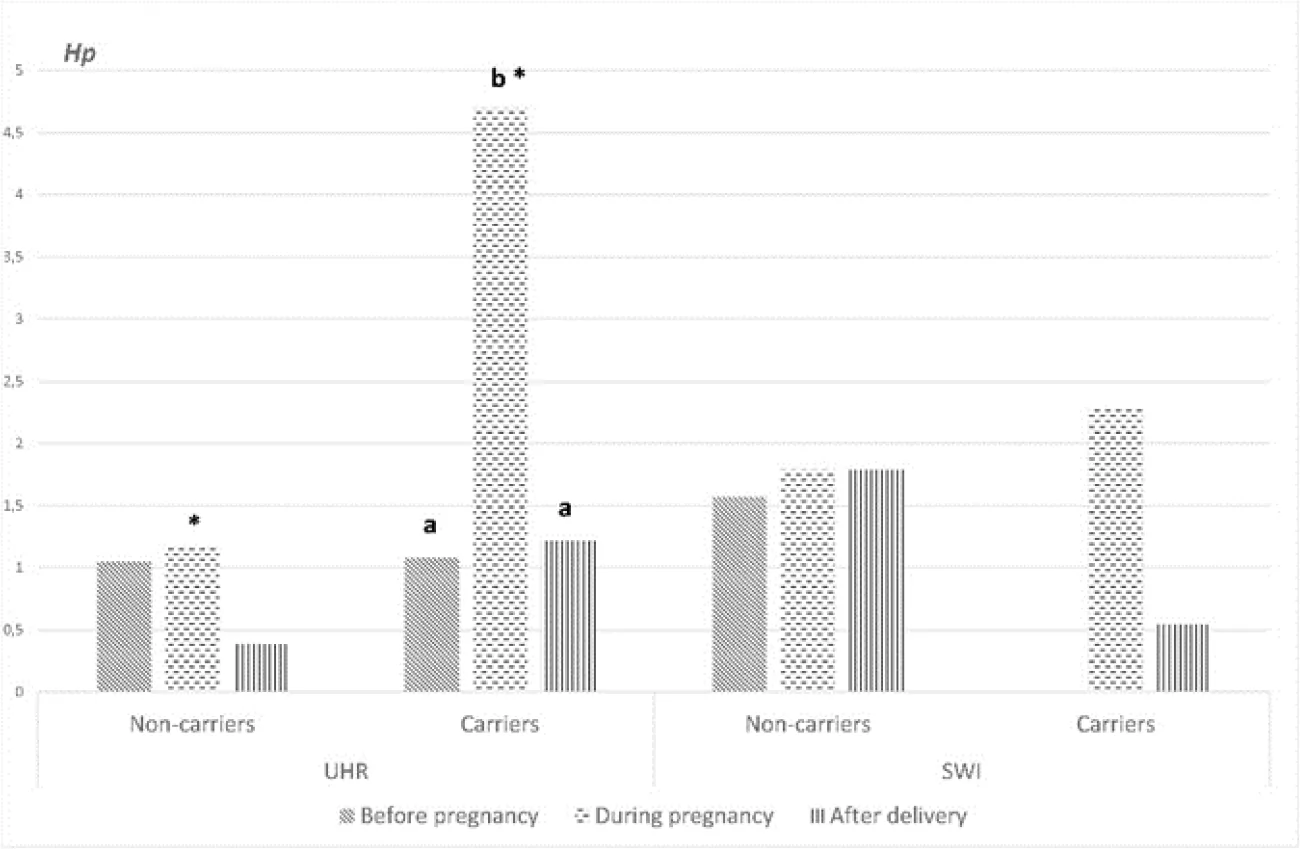
*Hp* gene expression at different reproductive stages in Uhruska (UHR) and Świniarka (SWI) ewes of parity two at study entry. Values represent the mean. ^a, b^ – means within each three-reproductive-stage group of bars with different subscript letters are significantly different (P-value < 0.05); * – means of non-carriers are significantly different to means of carriers (P-value < 0.05)

**Fig. 7.**
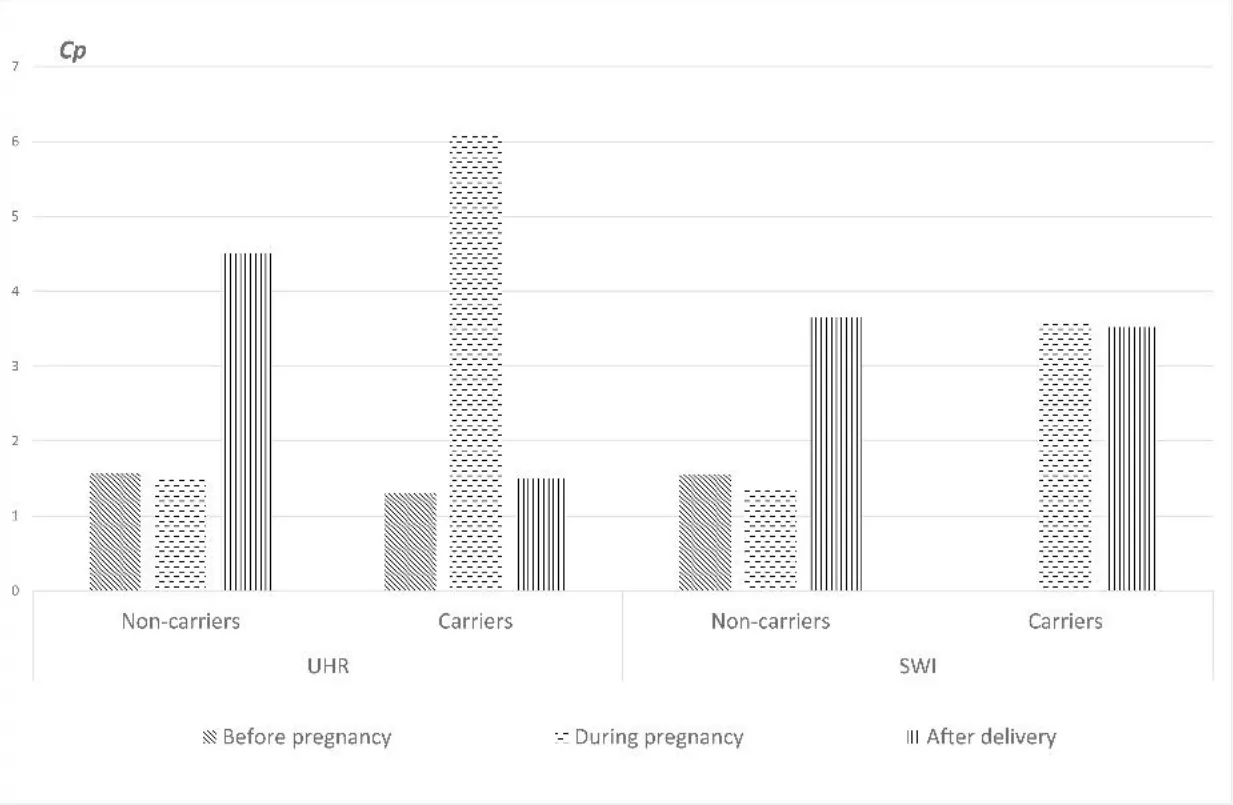
*Cp* gene expression at different reproductive stages in Uhruska (UHR) and Świniarka (SWI) ewes of parity two at study entry. Values represent the mean. ^a, b^ – means within each three-reproductive-stage group of bars with different subscript letters are significantly different (P-value < 0.05); * – means of non-carriers are significantly different to means of carriers (P-value < 0.05)

## Discussion

*Staphylococcus* is a broad genus of Gram-positive cocci that normally colonise the skin and mucous membranes of humans and animals but can also cause numerous infections. Among these cocci, the species *S. aureus* is the most problematic (12). Our current study, as well as our previous ones (9), confirmed that Uhruska and Świniarka sheep are reservoirs of *S. aureus* bacteria. However, *S. aureus* being present in the Świniarka breed in the perinatal period may result from the physiological effect of immunosuppression. In a previous study conducted by Greguła-Kania *et al*. (6), changes in white blood cell parameters were observed (the number of leucocytes and the percentage of lymphocytes decreased while the number of granulocytes increased) and had an impact on the maintenance of the foetus and the effect of immunosuppression. However, suppression of cell-mediated immunity reduces the host's ability to eliminate opportunistic pathogens. Consequently, *Staphylococcus aureus*, which may already colonise the skin or mucosal surfaces without causing disease, can proliferate more readily and establish infection during pregnancy or the periparturient period. The Uhruska, as a refined breed, is more susceptible to *S. aureus* colonisation than the Świniarka breed, and the latter may possibly have greater resistance to infection (9).

The changes in the means of all haematological parameters in the blood of ewes in both groups were in physiological value ranges (16, 30). There were no statistically significant differences between groups. A study in goats also revealed no significant differences in most haematological parameters (specifically in RBC, HGB, WBC, MCV, MCH and MCHC) even between goats with and without clinical mastitis. This indicates that even clinical signs of infection such as mastitis do not always lead to significant changes in blood parameters (4). Therefore, staphylococcal infections may exert more pronounced local effects (*e.g*. in the udder) than bring systemic changes in blood counts, although the inflammatory response may manifest itself through other indicators (*e.g*. inflammatory markers and cytokine changes).

These other indicators were the noted expression levels of the selected cytokine and acute-phase protein genes, showing that even local carriage of *S. aureus* in ruminants can lead to a systemic immune response, manifested by increased concentrations of cytokines in the blood. These changes reflect the activation of the immune system (13). The functions of the cytokines encoded by the studied genes include regulation of inflammation, metabolism and response to infections. Interleukin 6 is secreted by macrophages and endothelial cells; it stimulates the production of acute-phase proteins in the liver and supports the differentiation of B and T lymphocytes, including Th17 cells. The Th17–IL-17 axis is the main defence against extracellular pathogens such as *S. aureus* in humans and animals, as it is responsible for mobilising neutrophils to the site of infection, strengthening the epithelial barrier and inducing antimicrobial peptides in the skin and mucous membranes (3, 20). It is worth emphasising that, in our studies, statistically significant increases of *IL6* and *Hp* gene expression were observed in carriers of *S. aureus* during pregnancy compared to the periods before pregnancy and after lambing in lactation.

The obtained results showed that the level of activity of selected immune mechanisms may be correlated with the animal breed, since significant increases in *IL6* and *Hp* gene expression were observed in the leucocytes of the Uhruska breed in the *S. aureus* carriers compared to the non-carriers, but only insignificant ones were encountered in the Świniarka breed carriers. The observed breed-dependent differences in *IL6* and *Hp* expression suggest that the immune response to *Staphylococcus aureus* colonisation is influenced by genetic background. Uhruska sheep exhibited a significantly stronger activation of inflammatory and acute-phase pathways following bacterial carriage, whereas Świniarka sheep showed only minor changes in gene expression. This may indicate that Świniarka sheep, as a primitive native breed, are able to maintain bacterial colonisation under tighter immunological control, thereby limiting excessive inflammatory activation. Conversely, the stronger inflammatory response observed in Uhruska sheep may reflect either greater immune stimulation resulting from bacterial colonisation.

Haptoglobin is synthesised in response to the proinflammatory cytokine IL-6. In cattle, haptoglobin mRNA expression increases in both the liver and the mammary gland after *S aureus* infection, and this increase is noticeable 48 h after infection (1). Most studies on the immune response of ruminants have analysed milk somatic cells or udder endothelial cells. In our analyses, we noted increased expression of *IL6* and *Hp* genes in the blood. When haptoglobin forms a complex with haemoglobin, it has been shown to strongly inhibit haem binding by the *S. aureus* iron-regulated surface determinant IsdH receptor. Haptoglobin prevents pathogens from acquiring iron from haemoglobin, which limits their growth and multiplication (19). *Staphylococcus aureus* infection stimulates the host’s immune response to a varying degree, and the degree may determine the intensity of inflammation. A study conducted by Gilbert *et al*. (5) provided important information on the bovine immune response. During infection induced by *S. aureus*, mainly the activator protein 1 and IL-17A pathways were activated, which indicated that infections tended to be chronic and subclinical. The weak activation of the immune response by *S. aureus* may allow the bacteria to evade elimination by the host immune system. Many studies have been conducted to elucidate the complex physiological and cellular processes that occur in response to pathogens, but unfortunately the protective mechanisms are still not entirely clear. It is also worth noting that studies on the expression of immune system genes in sheep, as our present study, are very scarce compared to studies on other species such as cattle and pigs, and the potential mechanisms of the correlation between the activation of inflammation processes and the presence of *S. aureus* are still not fully understood (25, 29). *Staphylococcus aureus* possesses numerous factors that interact with the host immune response, and its presence activates various defence mechanisms.

The results of our study on the expression level of selected cytokine and acute-phase protein genes have shown that even local carriage of *S. aureus* in ruminants can lead to a systemic immune response, manifested by increased expression of cytokines in the blood. These changes reflect the activation of the immune system. Statistically significant increases of *IL6* and *Hp* gene expression were observed in carriers of *S. aureus* during pregnancy.

Uhruska sheep exhibited a significantly stronger activation of inflammatory and acute-phase pathways following bacterial carriage, whereas Świniarka sheep showed only minor changes in gene expression. This may indicate that Świniarka sheep, as a primitive native breed, are able to maintain bacterial colonisation under tighter immunological control, thereby limiting excessive inflammatory activation. Conversely, the stronger inflammatory response observed in Uhruska sheep may reflect either greater immune stimulation resulting from bacterial colonisation.

## Conclusion

The expression level of selected cytokine and acute-phase protein genes have shown that even local carriage of *S. aureus* in small ruminants can lead to a systemic immune response, manifested by increased expression of *IL6* and *Hp* genes in leucocytes.
